# Antibiotic Treatment Attenuates Behavioral and Neurochemical Changes Induced by Exposure of Rats to Group A Streptococcal Antigen

**DOI:** 10.1371/journal.pone.0101257

**Published:** 2014-06-30

**Authors:** Dafna Lotan, Madeleine Cunningham, Daphna Joel

**Affiliations:** 1 School of Psychological Sciences and Sagol School of Neuroscience, Tel Aviv University, Tel Aviv, Israel; 2 Department of Microbiology and Immunology, University of Oklahoma Health Sciences Center, Oklahoma City, Oklahoma, United States of America; Centre national de la recherche scientifique, France

## Abstract

Post-streptococcal A (GAS) sequelae including movement and neuropsychiatric disorders have been associated with improvement in response to antibiotic therapy. Besides eradication of infection, the underlying basis of attenuation of neuropsychiatric symptoms following antibiotic treatment is not known. The aim of the present study was to test the efficacy of antibiotic treatment in a rat model of GAS-related neuropsychiatric disorders. In the model, rats were not infected but were exposed to GAS-antigen or to adjuvants only (Control rats) and treated continuously with the antibiotic ampicillin in their drinking water from the first day of GAS-antigen exposure. Two additional groups of rats (GAS and Control) did not receive ampicillin in their drinking water. Behavior of the four groups was assessed in the forced swim, marble burying and food manipulation assays. We assessed levels of D1 and D2 dopamine receptors and tyrosine hydroxylase in the prefrontal cortex and striatum, and IgG deposition in the prefrontal cortex, striatum and thalamus. Ampicillin treatment prevented emergence of the motor and some of the behavioral alterations induced by GAS-antigen exposure, reduced IgG deposition in the thalamus of GAS-exposed rats, and tended to attenuate the increase in the level of TH and D1 and D2 receptors in their striatum, without concomitantly reducing the level of sera anti-GAS antibodies. Our results reinforce the link between exposure to GAS antigen, dysfunction of central dopaminergic pathways and motor and behavioral alterations. Our data further show that some of these deleterious effects can be attenuated by antibiotic treatment, and supports the latter’s possible efficacy as a prophylactic treatment in GAS-related neuropsychiatric disorders.

## Introduction

Group A β-hemolytic streptococcal (GAS) infection can lead in susceptible individuals to the development of delayed nonsuppurative sequelae autoimmune disorders, such as acute post-streptococcal glomerulonephritis, streptococcal reactive arthritis and acute rheumatic fever (ARF) [1], [2]. The autoimmune response can also target the central nervous system, leading to neurological and psychiatric disorders, such as Sydenham’s chorea (SC), pediatric autoimmune neuropsychiatric disorders associated with streptococcus (PANDAS), obsessive-compulsive disorder (OCD), and Tourette’s syndrome (for review see: [3]). SC is the main neurological manifestation of ARF, appearing weeks to months after GAS infection, and is characterized by involuntary movements and neuropsychiatric disturbances, including obsessive-compulsive symptoms, tics, and emotional lability [4].

Although the exact mechanism of pathogenesis in GAS-related neuropsychiatric disorders is not yet clear, it has been hypothesized that GAS infection induces the production of antibodies against GAS and neuronal determinants, through the process of molecular mimicry (for reviews see: [5]–[7]). It has been demonstrated that anti-GAS antibodies can bind to different brain determinants, and may consequently lead to increased altered neurotransmitter release, resulting in neuropsychiatric symptoms [8], [9].

There is some evidence suggesting that continued antibiotic treatment throughout childhood may prevent or decrease recurrences of SC and other GAS-related neuropsychiatric disorders [5], [6], [10]. Yet current data are too scant to reach firm conclusions (see: [11] for a critical discussion of the literature). Moreover, it is currently not clear whether the prophylactic action of antibiotics is achieved by preventing GAS reinfections or by the effects of antibiotics on other bacteria or if the effects may be directly on the brain (for review see: [12]).

The aim of the present study was to assess the effects of antibiotic treatment in an animal model of GAS-related neuropsychiatric disorders. In this model, exposure of male Lewis rats to GAS antigen leads to a syndrome which resembles behavioral, pharmacological, immunological and neural characteristics of GAS-related neuropsychiatric disorders [13]. More specifically, GAS-exposed rats show increased compulsive-like behavior and motor disturbances, which are attenuated by pharmacological agents used to treat the corresponding symptoms in human patients [13]; Immunologically, IgG in sera obtained from GAS-exposed rats demonstrates strong immunoreactivity to neural tissue, to D1 and D2 dopamine receptors [13], [14] and to 5-HT2a and 5-HT2c serotonin receptors [14], and activates calcium/calmodulin dependent protein kinase II (CaM-KII) signaling [13], as has been found for IgG in sera obtained from SC and PANDAS patients [8], [9], [13]; Finally, dopamine and glutamate levels are altered in the frontal cortex and basal ganglia of GAS-exposed rats [13].

**Figure 1:**
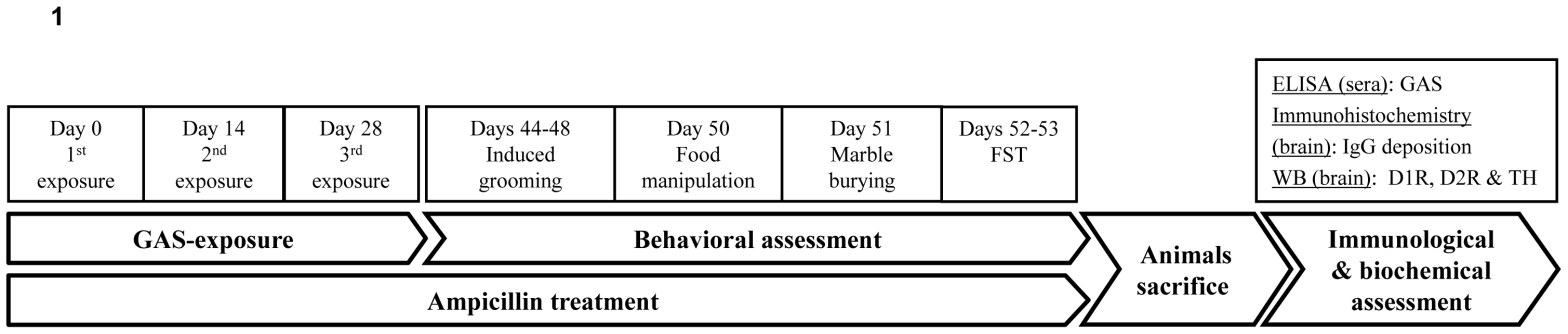
Experimental timeline. FST = forced swim test. WB = western blot. D1R = D1 dopamine receptor. D2R = D2 dopamine receptor. TH = tyrosine hydroxylase.

The present study used our rat model to assess the behavioral and biochemical effects of treatment with the β-lactam antibiotic ampicillin. Rats were exposed to GAS extract (GAS) or to adjuvants only (Control), and treated with ampicillin (given in their drinking water) (GAS-Ampicillin and Control-Ampicillin groups). Additional groups of GAS-exposed and Control rats received regular drinking water (GAS-Water and Control-Water groups). Motor abilities and compulsive- and depressive-like behaviors as well as the level of D1 and D2 dopamine receptors and of tyrosine hydroxylase in the prefrontal cortex (PFC) and striatum were assessed in rats from the four groups (See Figure 1 for details).

## Materials and Methods

### Ethics statement

The study was carried out according to institutional guidelines and approved by the Institutional Animal Care and Use Committee of Tel-Aviv University (P-11-014). All efforts were made to minimize animals suffering.

### Animals

Fifty eight Male Lewis rats (Harlan, Jerusalem, Israel), 5 weeks old, were housed 2–3 to a cage under a reversed 12-h light–dark cycle (lights on at 1900–0700 h) with ad libitum food and water (except for the duration of behavioral testing). Rats were weighed twice a week.

### Exposure to GAS

#### Streptococcus pyogenes

Protein type 18 (Manfraedo) GAS was obtained from Dr Allon Moses (Hadassah University Medical Center, Jerusalem, Israel) and grown as previously described [13]. Shortly, streptococci were grown in 400 mL Todd-Hewitt broth (HyLab, Rehovot, Israel) overnight at 37°C with rocking at 250 rpm. The next morning, the cells were collected by centrifugation at 5000 rpm for 15 min at 4°C. The cell pellets (in ∼1.5 gr) were stored frozen at −20°C until used.

#### Mutanolysin-Extracted GAS Antigen

A whole cell digest of M type 18 *Streptococcus pyogenes* was prepared as previously described [13]. Cell pellets were suspended in phosphate-buffered saline (PBS) containing mutanolysin (Sigma-Aldrich, Rehovot, Israel). Following incubation at 37°C for 2 h with rocking, the digest was further disrupted by sonication (Microson ultrasonic cell disruptor, Plainveiw, NY). The insoluble material was removed by centrifugation at 12,000×rpm (∼25000 g) for 30 min at 4°C. Protein concentration in the supernatant was determined using the Coomasie-Plus Bradford reagent (PIERCE) according to the supplier recommendations. The supernatant was dialyzed extensively against water (10,000 MWCO, Sigma-Aldrich, Rehovot, Israel) then lyophilized and the powder was stored at −70°C.

#### Exposure of rats to GAS antigen

The exposure protocol followed Brimberg et al. [13]. Twenty eight rats were handled for 2 min daily for 4 days before the beginning of the exposure protocol. The first exposure was done at 5 weeks of age. Before each injection, rats were lightly anaesthetized with Isoflorene (VetMarket, Petach Tikva, Israel). Each rat in the ***GAS group*** was immunized subcutaneously with 200 µl of 1∶1 emulsion of PBS containing 1.2 mg of the GAS antigen and Complete Freund’s adjuvant (CFA, Sigma-Aldrich, Rehovot, Israel) supplemented with 4 mg/ml of heat-killed mycobacteria H37RA (Difco Laboratories, Detroit, MI). In order to increase the permeability of the blood brain barrier [15] rats have received an intraperitoneal injection of 10^10^ heat-killed *Bordetella pertussis* (Bioport, Lansing, MI, USA) as an additional adjuvant. Two boosts were introduced two and four weeks following the first exposure. Each rat was boosted with 200 µl 1∶ 1 emulsion of incomplete Freund’s adjuvant (IFA, Sigma-Aldrich): PBS containing 1.2 mg of the GAS antigen. ***Control animals*** were injected with PBS and adjuvants only. Behavioral testing began when the rats were 11 weeks old.

### Antibiotic treatment

Rats were treated with the antibiotic ampicillin (VetMarket, Petach Tikva, Israel) in drinking water from the first day of GAS exposure throughout the entire experiment (a total of 7 weeks). Control and GAS-exposed rats in the ampicillin condition were given drinking water supplemented with ampicillin (1 mg/ml) in their home cages. Control and GAS-exposed rats in the Water condition were given regular drinking water.

### Behavioral Assessment

A 22-h food restriction schedule with water freely available was initiated at age 9 weeks. In all behavioral procedures the rater was blind to the animal’s experimental condition.

#### Food manipulation

Food manipulation was conducted as previously described [13]. One day following a 10 min exposure to a 38×21 cm Plexiglas observation box (habituation), food-deprived rats were placed in the box, and 5 min later several Purina rat food pellets (approximately 1g each) were introduced into the box. Rats were given 10 min to consume the food pellet, and their ability to manipulate it was rated independently by two observers, according to the scale of Kolb and Holmes [16]: 0, Unable to manipulate the food pellet with the forepaws; 1, Holds the food pellet against the floor with its forepaws when it eats; 2, Eats the food pellet from the floor and sometimes hold the food pellet in its forepaws; 3, Picks the food pellet up in its forepaws and partially eats it but drops the food pellet before it is all consumed; and 4, Sits up on its hind paws and holds the food pellet in its front paws until it is finished. Half scores were given when the animal behavior was in-between scale definitions. The observer rating behavior was blind to the animal condition.

#### Marble burying

Model burying was conducted as previously described [14]. Rats were placed individually in a cage measuring 37 cm long×21 cm wide×18 cm high, containing bedding that was 5 cm in depth, with nine marbles 2.3 cm in diameter arranged in two rows along the short wall of the cage. The number of buried marbles after 15 min was counted. Marbles were considered buried if they were at least one-half covered with bedding.

#### Forced swim test (FST)

Forced swim test was conducted as previously described [17]. Rats were placed in a vertical cylindrical glass container (50x20 cm) filled with tap water at 23°C ±1 (water depth was adjusted according to the rat’s weight). During the first session, they were placed in the water for 15 min. Twenty-four h later rats were again placed in the water for 5 min (test). The behaviors scored in the FST test were: (1) struggling - quick movements of the forelimbs such that the front paws break the surface of the water; (2) swimming - movement of forelimbs or hind limbs in a paddling fashion; and (3) immobility - floating with the absence of any movement [17].

#### Activity

Each rat was placed individually in a 38×21 cm plexiglas box located in a quiet room and allowed ten min of free exploration. Rats were videotaped using a camera located above each box for later activity evaluation. Activity level was coded and analyzed using the Biobserve software, by measuring the distance the rat completed in the ten min of exploration.

### Immunological and biochemical assessments

#### ELISA

Immunoreactivity to the GAS mutanolysin extracted streptococcal antigen was assessed using ELISA, as previously described [13]. Ninety six well ELISA plates (Nunc) were coated with 5 µg/ml of GAS mutanolysin extracted streptococcal antigen in PBS overnight at 4°C. Plates were blocked with 300 µl/well of 2% non-fat milk in PBS for 1 h at RT. Diluted rat serum was applied onto the plates in a dilution series (1∶500, 1∶2,500, and 1∶125,000) in 0.05% PBST and incubated for 1 h at RT. Following incubation, the plates were washed X3 with PBST. HRP conjugated donkey anti-rat antibodies (Jackson Immunolaboratories, USA, 1∶10,000 dilution in PBST) were added to the wells (100 µl/well) for 1 h at RT, followed by X3 washes with PBST. The Plates were developed using the chromogenic HRP substrate TMB (Sigma-Aldrich, Rehovot, Israel) (100 µl/well) and color development was terminated with 1 M H_2_SO_4_ (50 µl/well). The plates were read at 450 nm.

#### Western blot

Rats were overdosed with 100 mg/kg sodium pentobarbital, i.p, and perfused intracardially with cold perfusion buffer. Brains were removed, dissected and frozen at −80°C. The PFC and striatum were weighed and expelled into 500 µl RIPA buffer, containing protease inhibitor cocktail (Sigma), Sodium orthovanadate (1 mM), phenylmethylsulfonyl fluoride (PMSF, 1 mM) and Dithiothreitol (DTT, 1 mM). Tissue was homogenized briefly and the homogenates were centrifuged at 1500×g for 15 min at 4°C, after which the supernatants were collected, and protein content was determined using Pierce BCA protein assay kit (Thermo) and Bovine serum albumin as a standard protein (Thermo) and an ELISA reader. Samples (12–15 µg total protein) were electrophoresed on 10% SDS- polyacrylamide gel (BioRad Laboratories). Proteins were transferred onto nitrocellulose membranes (Tamar Laboratories Supplies) for immunoblotting using constant voltage (100 mv) for 1.5 h. A cooling coil was used to prevent excessive heating. Membranes were blocked in western blocker solution (Sigma) for 1 h prior and incubated with anti- D1 dopamine receptor (1∶500, Millipore; Cat # ABN20), anti- D2 dopamine receptor (1∶1000, Millipore; Cat # AB5084P), and anti- tyrosine hydroxylase (TH, 1∶1000, Millipore; Cat # MAB318) antibodies in TBST overnight at 4°C. The membranes were then incubated for 1 h with horseradish peroxidase- conjugated anti-rabbit IgG or anti-mouse IgG secondary antibodies (1∶10,000, Jackson Immunolaboratories, USA), and proteins were detected using chemiluminescence detecting substrate (Biological Industries). Western blotting for actin served as a loading control. Analysis and quantification of the bands was performed using ImageJ software. D1, D2 and TH density was divided by the corresponding actin density, and the relative density was normalized against that of the control group.

#### Preparation of tissue sections

Rats were overdosed with 100 mg/kg sodium pentobarbital, i.p, and perfused intracardially with cold perfusion buffer. Brains were removed and placed in 4% paraformaldehyde over night, after which they were cryoprotected in 30% sucrose solution for at least 48 h. For immunostaining, brains were sectioned in the horizontal plane at 16 µm for detecting IgG deposits, and sections were mounted on gelatin-coated slides. Slides were stored at −70°C.

#### Immunostaining for IgG deposits

To assess IgG deposits in the brain, 16 µm sections were incubated in PBS until reaching room temperature, and rinsed for 30 min with 3% H_2_O_2_ in methanol, followed by X2 washes with PBS and X1 wash in PBST. Following a 1 h blocking (20% normal goat serum in PBST) at RT, sections were incubated for 1 h in biotinylated secondary anti-rat IgG (Vector Laboratories, Burlingame, USA) diluted in 2% normal goat serum in PBST. Following X3 PBST washes, equal number of GAS and control rats’ sections were developed using Vector ABC Elite Kit and diaminobenzidine (DAB) solution (Vector Laboratories, Burlingame, USA). The immunostained brain sections were viewed and photographed using light microscope and Nikon NIS imaging software. Immunostaining was assessed by viewing the sections microscopically and determining whether the relevant area showed antibody (IgG) staining or not.

### Statistical Analysis

Analysis of variance (ANOVA) with main factors of Exposure and Treatment were performed on food manipulation scores, number of marbles buried, immobility time in the FST, anti-GAS antibody levels in sera, and D1, D2 and TH levels in the striatum and PFC. Significant effects were followed by post-hoc least significant difference (LSD) analyses (the specific analyses are detailed in the Results section). We considered values as significant when p<0.05. All data are presented as means ± SEM.

## Results

### Behavioral

#### Ampicillin treatment attenuated the deleterious effects of GAS-exposure in the food manipulation, marble burying and forced swim tests (FST)

**Figure 2:**
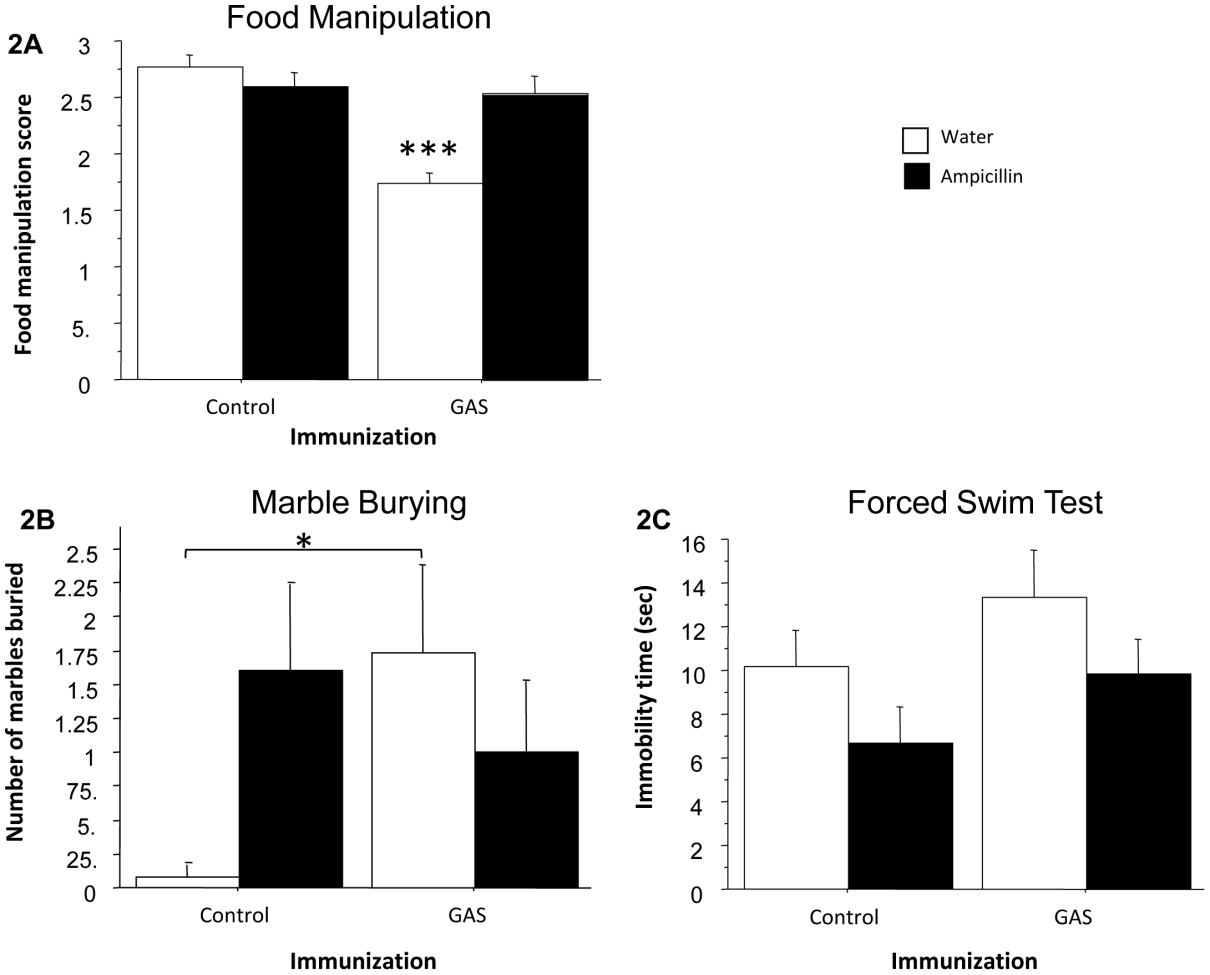
Behavioral effects of GAS exposure and ampicillin treatment. Effects of streptococcal exposure and ampicillin treatment on (A) food manipulation, (B) marble burying, and (C) forced swim test (FST). (A) The mean and SE of food manipulation scores of GAS-Water, Control-Water, GAS-ampicillin, and Control-ampicillin rats. (B) The mean and SE of number of marbles buried by GAS-Water, Control-Water, GAS-ampicillin, and Control-ampicillin rats. (C) The mean and SE of the duration of immobility time in the FST of GAS-Water, Control-Water, GAS-ampicillin, and Control-ampicillin rats. N per group  = 12–15 rats. *p<0.05, ***p<0.0001.

Figure 2 presents food manipulation scores (Fig. 2A), number of marbles buried in the marble burying test (Fig. 2B) and immobility time in the FST (Fig. 2C), of GAS-exposed rats and adjuvant-exposed control rats treated with ampicillin or water. As can be seen, GAS rats were impaired in manipulating food, and ampicillin treatment prevented this impairment (Fig. 2A, ANOVA: Exposure, F(1,54) = 20.262, p<0.0001; Treatment, F(1,54) = 6.632, p<0.05; Exposure × Treatment interaction, F(1,54) = 15.658, p<0.0005, see Figure for the results of the post hoc analysis). GAS-exposed rats buried more marbles in the marble burying test compared to Control rats. Ampicillin treatment had opposite effects on marble burying in GAS-exposed and Control rats, tending to decrease and increase it, respectively (Fig. 2B, ANOVA: a significant Exposure × Treatment interaction only, F(1,53) = 4.056, p<0.05, see Figure for the results of the post hoc analysis). In the FST, ampicillin treatment tended to decrease immobility time in both GAS and control rats (Fig. 2C, ANOVA: the effect of Treatment approached significance, F(1,52) = 3.767, p = 0.0577). No differences were found in activity level between the four groups (all p’s >0.25, data not shown).

### Biochemical

#### GAS Rat Sera Reacted with Streptococcal Antigens

**Figure 3:**
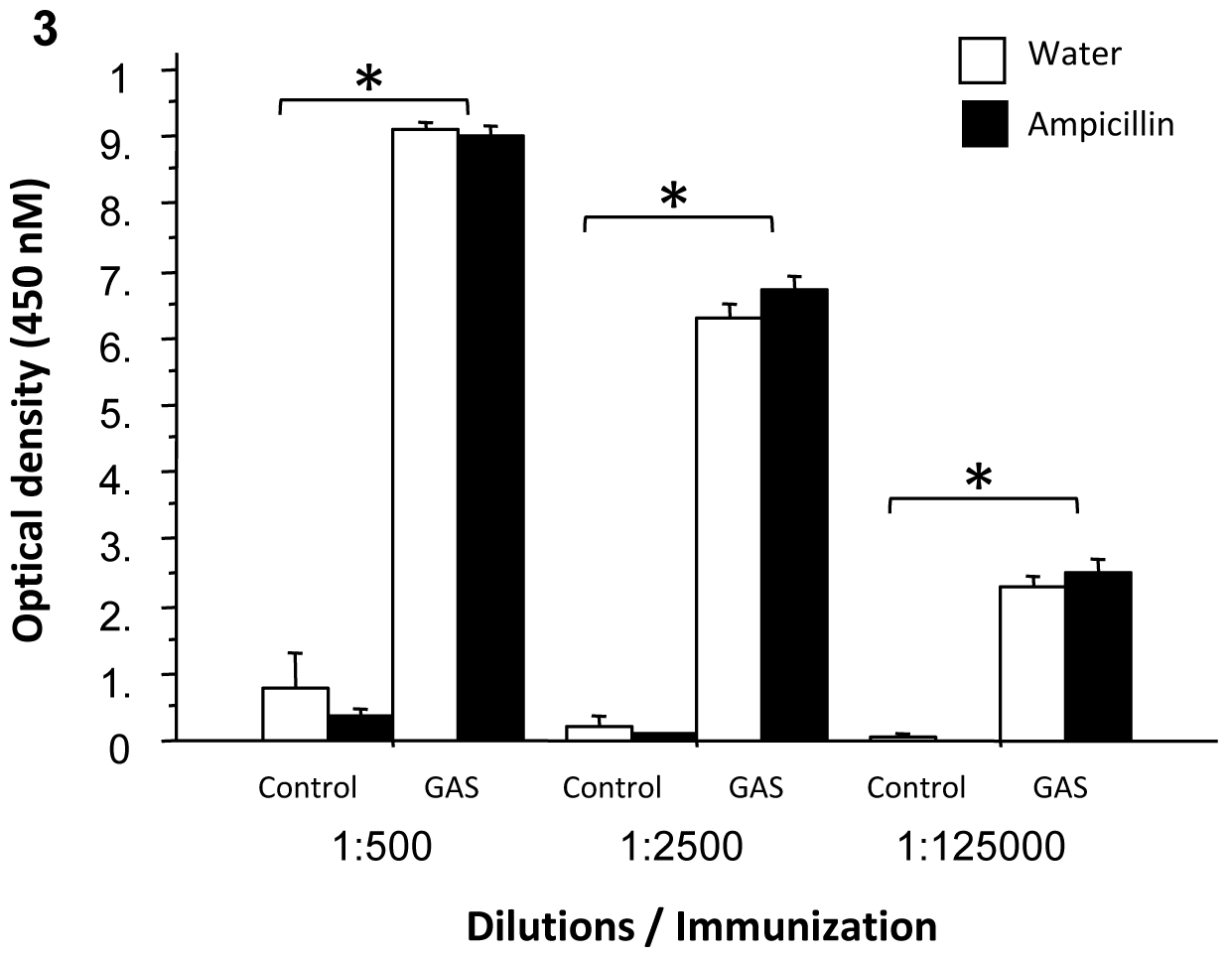
Immunological effects of GAS exposure and ampicillin treatment. Effects of streptococcal exposure and ampicillin treatment on immunoractivity against GAS mutanolysin extracted antigen, of sera taken from GAS-Water, Control-Water, GAS-ampicillin, and Control-ampicillin rats. N per group = 13–15 rats. *p<0.0001.

IgG antibodies in sera of GAS-exposed rats were significantly elevated against the GAS cell wall antigen, the immunogen. Ampicillin treatment did not affect anti-streptococcal antibody IgG levels in the serum of immunized rats (Fig. 3, ANOVA: a significant effect of Exposure only, F(1,54) = 533.885, p = 0<0.0001).

#### Changes in D1 and D2 dopamine receptors and tyrosine hydroxylase levels in the brain following exposure to GAS and ampicillin treatment

**Figure 4:**
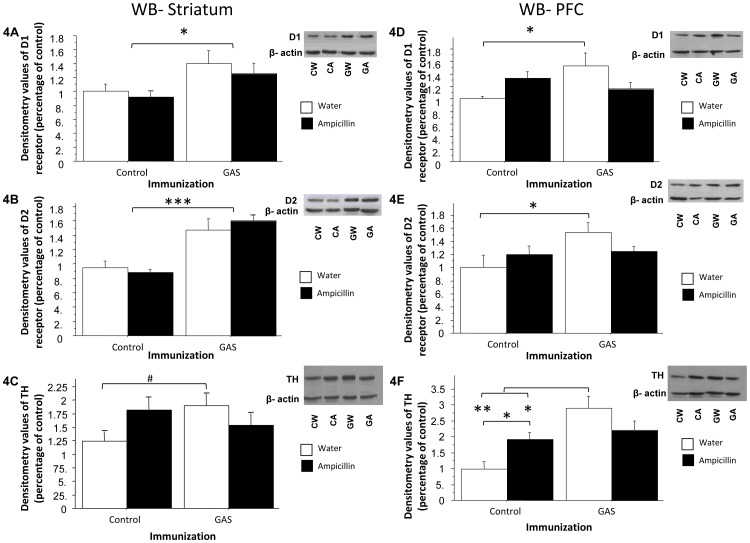
Biochemical effects of GAS exposure and ampicillin treatment. Effects of streptococcal exposure and ampicillin treatment on D1 (A, D) and D2 (B, E) dopamine receptors and TH levels (C, F) in the striatum (A–C) and PFC (D–F) of GAS-exposed and control rats treated with ampicillin or water. The densitometry value of each of the three proteins was divided by the densitometry value of actin, and this ratio was divided by the mean protein/actin ratio of the GAS-Water group. N per group = 5–7 rats. CW = Control-Water; CA = Control-Ampicillin; GW = GAS-Water; GA = GAS-Ampicillin. *p<0.05, **p<0.0005, ***p<0.0001, #p = 0.053.

Figure 4 presents the levels of D1 and D2 dopamine receptors and TH in the striatum and PFC of GAS-exposed and Control rats treated with ampicillin or water. **In the striatum,** D1 and D2 dopamine receptor levels were significantly higher in GAS-exposed rats compared to Control rats and ampicillin treatment had no effect (**D1:** Fig. 4A, ANOVA: a significant effect of Exposure only, F(1,20) = 6.712, p<0.05; **D2:** Fig. 4B, ANOVA: a significant effect of Exposure only, F(1,20) = 34.944, p<0.0001). TH levels were higher in GAS-exposed rats compared to control rats, and ampicillin treatment had opposite effects on TH levels, tending to decrease and increase it, respectively (Fig. 4C, ANOVA: a significant Exposure × Treatment interaction only, F(1,22) = 4.609, p<0.05, see Figure for the results of the post hoc analysis). **In the PFC**, D1 dopamine receptor levels were significantly increased in the GAS-exposed group compared to the Control group, and ampicillin treatment had opposite effects on these levels, tending to decrease and increase it, respectively (Fig. 4D, ANOVA: a significant Exposure × Treatment interaction only, F(1,21) = 7.018, p<0.05, see Figure for the results of the post hoc analysis). The levels of D2 dopamine receptors in the PFC were also elevated in the GAS-exposed group compared to the Control group, but the seemingly opposite effect of ampicillin on these levels failed to reached significance (Fig. 4E, ANOVA: a significant effect of Exposure, F(1,22) = 4.465, p<0.05; Exposure × Treatment interaction, F(1,22) = 3.072, p = 0.0936, see Figure for the results of the post hoc analysis). TH levels in the PFC were significantly increased in the GAS-exposed group compared to the Control group, and ampicillin treatment had opposite effects on these levels, tending to decrease and increase it, respectively (Fig. 4F, ANOVA: significant effect of Exposure, F(1,22) = 14.653, p<0.001, and Exposure × Treatment interaction, F(1,22) = 8.037, p<0.01, see Figure for the results of the post hoc analysis).

#### Ampicillin treatment reduced IgG deposition in the thalamus of GAS-exposed rats, but not in PFC and striatum

**Figure 5:**
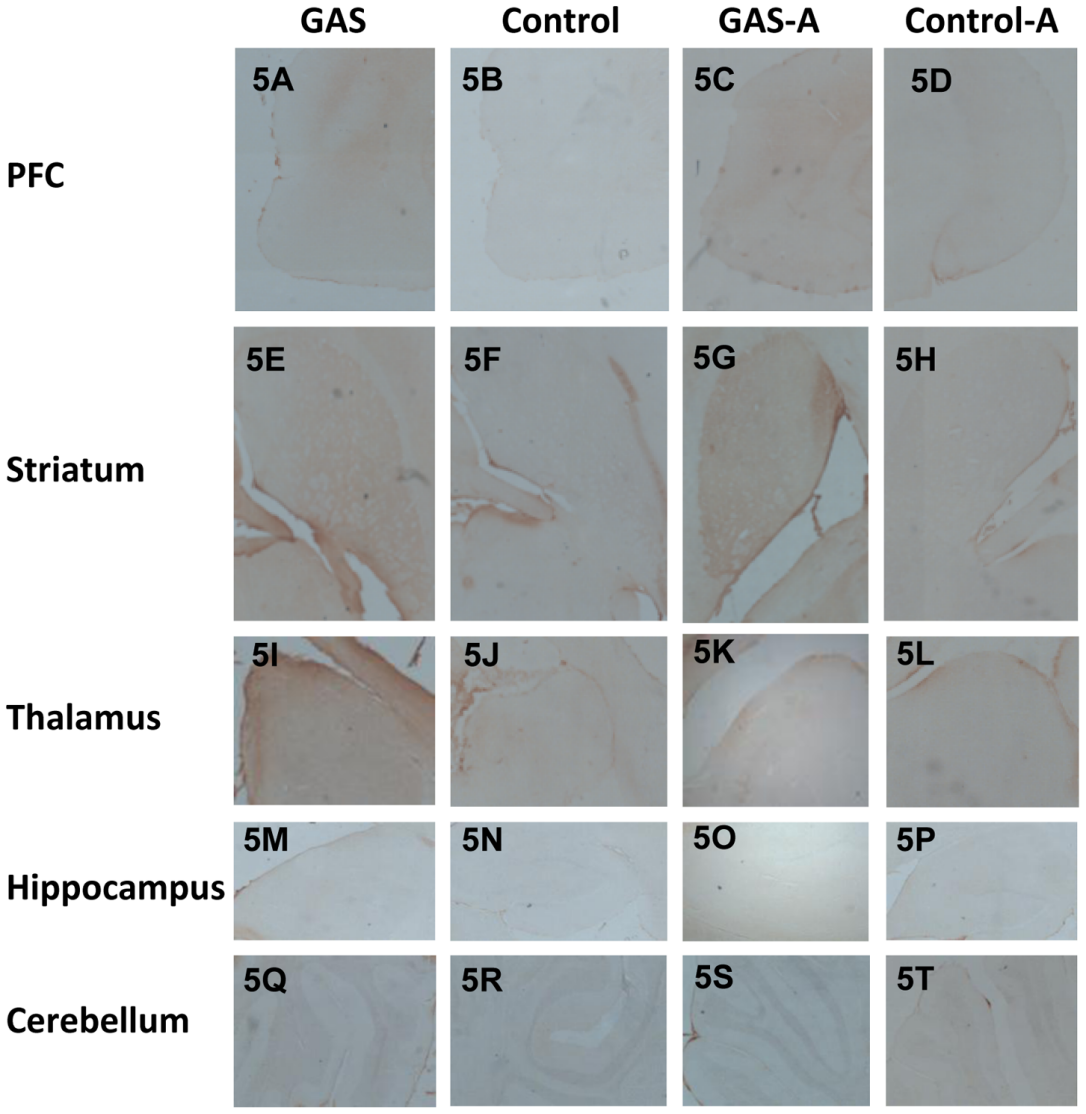
Immunoreactivity of IgG with the brain following GAS exposure and ampicillin treatment (low resolution). IgG deposition in brains of GAS-exposed and control rats treated with ampicillin or water. IgG deposits in brain sections taken through the PFC (A–D), striatum (E–H), thalamus (I–L), hippocampus (M–P), and cerebellum (Q–T) of a GAS-Water (A, E, I, M, Q), Control-Water (B, F, J, N, R), GAS-ampicillin (C, G, K, O, S) and Control-ampicillin (D, H, L, P, T) rat, at a magnification ×4. Tissue sections were incubated in biotinylated-anti-rat IgG and then incubated with avidin using Vectastain ABC kit (Vector Laboratories, Burlingame, CA, USA). Anti-IgG binding to brain sections was detected using diaminobenzidine for visualization of antibody deposition. Scale bar = 200 µm.

**Figure 6:**
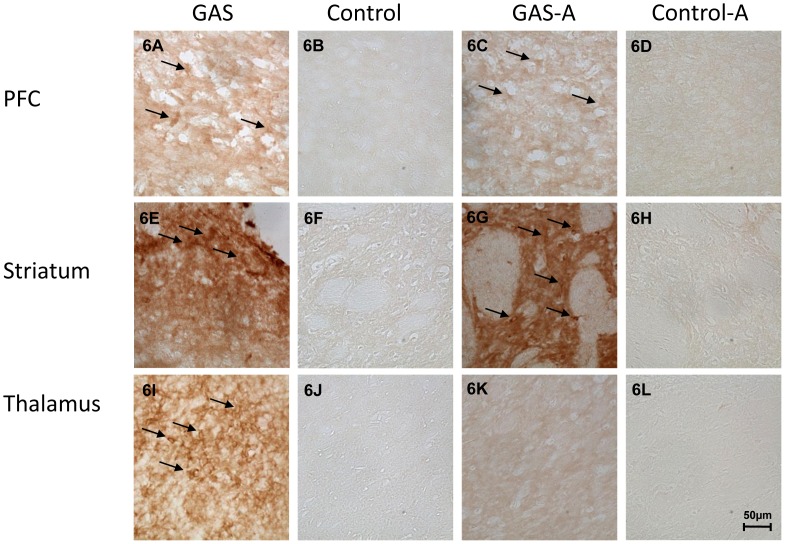
Immunoreactivity of IgG with the brain following GAS exposure and ampicillin treatment (high resolution). IgG deposition in brains of GAS-exposed and control rats treated with ampicillin or water. IgG deposits in brain sections taken through the PFC (A–D), striatum (E–H), and the thalamus (I–L), of a GAS-Water (A, E, I), Control-Water (B, F, J), GAS-ampicillin (C, G, K) and Control-ampicillin (D, H, L) rat, at a magnification ×40. Tissue sections were incubated in biotinylated-anti-rat IgG and then incubated with avidin using Vectastain ABC kit (Vector Laboratories, Burlingame, CA, USA). Anti-IgG binding to brain sections was detected using diaminobenzidine for visualization of antibody deposition. Arrows mark staining of neuronal cells. Scale bar = 50 µm.

IgG reactivity was detected in the striatum, thalamus, and PFC of GAS-exposed rats (Fig. 5A, E, I and 6A, E, I). Little or no IgG staining was seen in these brain areas in Control rats (Fig. 5B, F, J and 6B, F, J). Treatment with ampicillin reduced IgG deposition in the thalamus (Fig. 5K and 6K), but not in the striatum and PFC (Fig. 5C and G and 6C and G). No IgG depositions were found in the hippocampus and cerebellum (Fig. 5 M–T).

## Discussion

Our study evaluated the role of antibiotic treatment (ampicillin) in preventing the behavioral, immunological and neurochemical alterations caused by exposure of male Lewis rats to GAS antigen (See Table 1 for a summary of the findings). Our findings provide further support for a link between GAS exposure, dysfunction of the dopaminergic system and behavioral alterations and reveal that ampicillin treatment attenuates some of the behavioral and neurochemical changes that are induced by GAS exposure without concomitantly affecting the level of sera anti-GAS antibodies.

**Table 1:** Summary of the behavioral, immunological and biochemical results.

	Ampicillintreatment	GASexposure & Ampicillin	GASexposure
**Behavioral**			
Food manipulation	–	↓	–
Marble burying	∼ ↑	↑	–
Immobility in FST	∼ ↓	–	–
Activity	–	–	–
**Immunological**			
Anti-GAS antibodies in sera	–	↑	↑
**Immunohistochemistry**			
IgG deposits in the:			
Striatum	–	↑	↑
PFC	–	↑	↑
Thalamus	–	↑	–
**Biochemical**			
Changes in striatal levels of:			
D1 receptor	–	↑	↑
D2 receptor	–	↑	↑
Tyrosine hydroxylase	∼ ↑	↑	–
Changes in PFC levels of:			
D1 receptor	–	↑	–
D2 receptor	–	↑	–
Tyrosine hydroxylase	↑	↑	↑

– : No effect.

↑ : Increase.

↓ : Decrease.

∼ : tendency.

All signs relate to a comparison to the Control-Water group.

### Exposure to GAS

The present study replicated and extended our previous findings regarding the effects of exposure of rats to GAS extract [13], [14]. Immunologically, exposure to GAS led to an increase in sera anti-GAS antibodies as well as to the presence of antibodies in the striatum, PFC and thalamus, as we have previously found [13]. Behaviorally, GAS exposure led to impaired food manipulation [13], [14] and increased marble-burying without a concomitant increase in activity level. These behavioral alterations may be relevant to symptoms commonly observed in GAS-related neuropsychiatric disorders, including impaired fine motor control [6], [18], [19], anxiety [19]–[22] and compulsions [18], [20], [22], [23].

A novel finding of the present study is that GAS-exposure led to an increase in the level of TH and of D1 and D2 dopamine receptors in the striatum and PFC. These results add to our previous findings of altered dopamine function following GAS exposure [13], [14]. Specifically, the D2 antagonist, haloperidol, ameliorated motor impairment in GAS-exposed rats [13]; anti-D1 and anti-D2 IgG were detected in the sera of these rats [13], [14]; and their dopamine level in the frontal cortex and basal ganglia was increased [13]. Involvement of the dopaminergic system has been repeatedly implicated in the pathogenesis of GAS-related neuropsychiatric disorders: Anti-dopaminergic drugs such as haloperidol are used to treat motor symptoms in GAS-related disorders [11], [19]; anti-D1 and anti-D2 antibodies were detected in the sera of SC and PANDAS patients [13], [24]; and increased levels of TH were found in brains of rats injected with antibodies purified from SC patients [25]. Interestingly, sera and IgG from SC and PANDAS patients [8], [9] and from GAS-exposed rats [13] led to increased CAM-KII activity in vitro, and CAM-KII activity has been shown to up-regulate the D2 dopamine receptor promoter in vitro [26].

There are previous reports that antibodies to neuronal cell surface can lead to changes in the function and level of their targeted protein, thus leading to neuronal and behavioral alterations. For instance, anti-AMPA receptor antibodies in AMPA receptor encephalitis as well as anti-NMDA receptor antibodies in anti-NMDA receptor encephalitis and in para-neoplastic syndrome were reported to decrease synaptic density of the receptor [27], [28], and anti-muscarinic receptor antibodies were found to up-regulate the gene expression of the targeted receptor [29]. Although the mechanism by which antibodies may alter receptor levels in the brain is currently unknown, several possibilities have been raised, including binding of antibodies to the receptors and modulation of their levels by agonism/antagonism of the receptor, internalization of the receptor, and blockade of cellular interactions with different intra- or extra-cellular molecules or with other cells (for review see: [30]).

#### Linking GAS exposure, dopamine dysfunction and altered behavior

Taken together, the findings in humans and rats suggest one possible mechanism by which exposure to GAS may lead to altered functioning of the dopaminergic system and to altered behavior. Specifically, exposure to GAS may induce auto-antibodies that bind to D1 and D2 dopamine receptors consequently leading to upregulation of these receptors, to an increase in TH content, and to behavioral symptoms. In support of the direct role of antibodies in the induction of neural and behavioral abnormalities, we have recently shown that passive transfer of antibodies purified from GAS-exposed rats’ sera into the brain of otherwise intact rats was sufficient to induce behavioral abnormalities in these rats [14], and Doyle et al. [31] have recently shown that antibodies purified from SC patients and infused into the striatum of rats caused an up-regulation of dopaminergic activity and motor alterations.

### Treatment with ampicillin

Ampicillin treatment eliminated the impairment in food manipulation, and tended to attenuate the increased marble burying in GAS-exposed rats. Immunologically, ampicillin treatment had no effect on the level of sera anti-GAS antibodies, but reduced IgG deposition in the thalamus (but not in the PFC and striatum) of GAS-exposed rats. Neurochemically, ampicillin treatment tended to attenuate the increase in the level of TH and of D1 receptors in the PFC and of TH in the striatum of GAS-exposed rats.

The finding that ampicillin treatment reduces some of the deleterious behavioral effects of exposure to GAS is in line with the clinical observation that antibiotic treatment throughout childhood is effective in reducing relapse of SC [5], [10]. In the rat model, antibiotic treatment was effective even though there are no live GAS bacteria, and was not dependent on a reduction in the level of anti-GAS IgG in the sera. Interestingly, in the clinic, antibiotic treatment has been reported to be effective even in patients with low bacterial titers, suggesting that antibiotics may act on more than just the throat bacteria [6].

The prophylactic action of antibiotics may be achieved by their immunomodulatory effects [32] or by their neuroprotective effects. Evidence for the latter arises from animal models of amyotrophic lateral sclerosis [33], traumatic brain injury [34], [35], multiple sclerosis [36], Huntington’s disease [37], [38], stroke [39], and Parkinson’s disease [40]. Such neuroprotective effects may be mediated by the immunomodulatory effects of these drugs [39] or by a direct regulation of different brain proteins [33], [41], [42]. Interestingly, the present study found that ampicillin treatment to control rats led to increased TH levels in the PFC, and tended to increase TH level in the striatum and D1 and D2 levels in the PFC. Both the immunomodulatory and the neuroprotective effects of antibiotics may be achieved directly or by their effects on the GI microbiota, which is essential for the normal development and functioning of the host immune system [43]–[47], as well as brain development and function [48], [49]. Previous studies in animals have shown that introduction of specific bacteria or elimination of others can lead to behavioral and neural changes, including among others, changes in depressive- and anxiety-like behaviors and alterations in the production, release and metabolism of neurotransmitters and the expression of receptors (for review see: [50]). Interestingly, a recent study has demonstrated that introduction of the gram negative bacteria B. fragilis to a mice model of autism spectrum disorders ameliorated deficits in communicative, stereotypic, sensorimotor, and anxiety-like behaviors, including a reduction in marble burying [51].

Although the present study does not reveal the mechanism(s) by which ampicillin treatment achieved its beneficial effects, some insight into the relations between the neurochemical and behavioral effects of ampicillin treatment may be gained from comparing the pattern of these effects in GAS-exposed and in control rats. Specifically, there is an interesting parallel between the opposite effects of ampicillin on marble burying and on the level of D1 dopamine receptors and of TH in the PFC and striatum of GAS-exposed and control rats (i.e., ampicillin treatment decreased the behavioral and neurochemical measures in GAS rats and increased them in control rats). This parallel may suggest that the increase in these neurochemical measures in GAS-exposed rats caused the increased marble burying, and their attenuation by ampicillin treatment led to the prevention of the behavioral change. Indeed, previous studies have found that manipulations leading to a reduction in dopamine levels in the PFC or striatum led to reduced marble burying [52], [53], although other studies found the opposite effect [54], [55]. Of the immunological and neurochemical measures that were taken in the present study, IgG deposits in the thalamus are the only one which parallels the pattern of behavioral deficits in the food manipulation task (i.e., no IgG deposits – no motor deficit). In humans, damage to the thalamus caused by stroke can lead to choreic movements [56], [57] and thalamic structural and functional alterations are correlated with motor symptoms in disorders such as SC, TS, Huntington’s disease, Parkinson’s disease and schizophrenia [58]–[63]. In animals, different manipulations that disrupt thalamic functioning lead to motor alterations such as reduced grip strength, involuntary clasping movement and impairments in limb coordination and balance [64], [65]. Moreover, Hansen et al. [66] found that intra-ventricular injection of antibodies purified from patients suffering from the stiff person syndrome were localized to the thalamus of the injected rats and reduced forelimb grip strength.

In summary, our results reinforce the link between exposure to GAS antigen, dysfunction of central dopaminergic pathways and motor and behavioral alterations [13], and suggest that some of these deleterious effects can be attenuated by antibiotic treatment, independently of the latter’s direct impact on GAS.
